# DOCK2 Deficiency Causes Defects in Antiviral T-Cell Responses and Impaired Control of Herpes Simplex Virus Infection

**DOI:** 10.1093/infdis/jiae077

**Published:** 2024-02-15

**Authors:** Katrina L Randall, Inge E A Flesch, Yan Mei, Lisa A Miosge, Racheal Aye, Zhijia Yu, Heather Domaschenz, Natasha A Hollett, Tiffany A Russell, Tijana Stefanovic, Yik Chun Wong, Sandali Seneviratne, Fiona Ballard, Raquel Hernandez Gallardo, Sarah N Croft, Christopher C Goodnow, Edward M Bertram, Anselm Enders, David C Tscharke

**Affiliations:** Division of Immunology and Infectious Diseases, John Curtin School of Medical Research, The Australian National University, Canberra, ACT, Australia; School of Medicine and Psychology, The Australian National University, Canberra, ACT, Australia; Division of Immunology and Infectious Diseases, John Curtin School of Medical Research, The Australian National University, Canberra, ACT, Australia; Division of Immunology and Infectious Diseases, John Curtin School of Medical Research, The Australian National University, Canberra, ACT, Australia; Division of Immunology and Infectious Diseases, John Curtin School of Medical Research, The Australian National University, Canberra, ACT, Australia; Division of Immunology and Infectious Diseases, John Curtin School of Medical Research, The Australian National University, Canberra, ACT, Australia; Division of Immunology and Infectious Diseases, John Curtin School of Medical Research, The Australian National University, Canberra, ACT, Australia; Division of Immunology and Infectious Diseases, John Curtin School of Medical Research, The Australian National University, Canberra, ACT, Australia; Division of Immunology and Infectious Diseases, John Curtin School of Medical Research, The Australian National University, Canberra, ACT, Australia; Division of Immunology and Infectious Diseases, John Curtin School of Medical Research, The Australian National University, Canberra, ACT, Australia; Division of Immunology and Infectious Diseases, John Curtin School of Medical Research, The Australian National University, Canberra, ACT, Australia; Division of Immunology and Infectious Diseases, John Curtin School of Medical Research, The Australian National University, Canberra, ACT, Australia; Division of Immunology and Infectious Diseases, John Curtin School of Medical Research, The Australian National University, Canberra, ACT, Australia; Division of Immunology and Infectious Diseases, John Curtin School of Medical Research, The Australian National University, Canberra, ACT, Australia; Division of Immunology and Infectious Diseases, John Curtin School of Medical Research, The Australian National University, Canberra, ACT, Australia; Division of Immunology and Infectious Diseases, John Curtin School of Medical Research, The Australian National University, Canberra, ACT, Australia; Division of Immunology and Infectious Diseases, John Curtin School of Medical Research, The Australian National University, Canberra, ACT, Australia; Garvan Institute of Medical Research, University of New South Wales, Darlinghurst, NSW, Australia; Division of Immunology and Infectious Diseases, John Curtin School of Medical Research, The Australian National University, Canberra, ACT, Australia; Division of Immunology and Infectious Diseases, John Curtin School of Medical Research, The Australian National University, Canberra, ACT, Australia; Division of Immunology and Infectious Diseases, John Curtin School of Medical Research, The Australian National University, Canberra, ACT, Australia

**Keywords:** dedicator of cytokinesis 2, DOCK2, herpes simplex virus, T-cell activation, viral control

## Abstract

The expanding number of rare immunodeficiency syndromes offers an opportunity to understand key genes that support immune defense against infectious diseases. However, analysis of these in patients is complicated by their treatments and comorbid infections, requiring the use of mouse models for detailed investigations. We developed a mouse model of DOCK2 immunodeficiency and herein demonstrate that these mice have delayed clearance of herpes simplex virus type 1 (HSV-1) infections. We also uncovered a critical, cell-intrinsic role of DOCK2 in the priming of antiviral CD8^+^ T cells and in particular their initial expansion, despite apparently normal early activation of these cells. When this defect was overcome by priming in vitro, DOCK2-deficient CD8^+^ T cells were surprisingly protective against HSV-1 disease, albeit not as effectively as wild-type cells. These results shed light on a cellular deficiency that is likely to impact antiviral immunity in DOCK2-deficient patients.

## Teaser

DOCK2 deficiency was explored in a model of herpes simplex virus infection and immunity, finding a critical role for this protein in antiviral CD8^+^ T-cell priming and initial expansion.

Dedicator of cytokinesis 2 (DOCK2) immunodeficiency is a disease that leads to severe immunocompromise, being fatal in 2 of the original 5 cases described, and requiring bone marrow transplantation in the other cases [1]. Patients with mutations in *DOCK2* present with combined immunodeficiency with early-onset invasive bacterial and viral infections [1]. These include infections caused by herpesviruses (varicella zoster virus and cytomegalovirus), adenovirus, and mumps, as well as bacterial infections and a likely case of *Pneumocystis jirovecii* [1–4].

DOCK2 is a member of the DOCK family of guanine nucleotide exchange factors (GEFs) and has been previously shown in mice to be a GEF for RAC1, a GTPase that regulates actin organization, cell signaling, and cell proliferation [5, 6]. DOCK2 is predominantly expressed in hematopoietic cells, particularly the cells of the immune system, but can be found at low levels in other tissues [7]. DOCK2 knockout mice have severe peripheral lymphopenia, due to defects in development and chemotaxis of T cells, and lymphoid follicle hypoplasia [7]. DOCK2 is also important for proper T-cell “synapse” formation upon engaging antigen-bearing cells and is required for the movement of the T-cell receptor (TCR) and lipid rafts into the contact area [5]. As a result, DOCK2-deficient T cells have reduced antigen-specific proliferation [5]. DOCK2 is also required for normal B-cell function, being important for integrin activation in response to chemokine signaling [8]. More recently, studies by Mahajan et al [9] have described a skewing toward memory T cells and away from naive T cells in the DOCK2*^hsd^* strain, indicating that mutations in DOCK2 are changing the signaling strength threshold for entry into this subset of cells.

While many roles for DOCK2 have been found, the majority of these were not explored in the context of infection, which is arguably the most relevant setting. The only such studies to date are for bacterial infection and found that DOCK2 is important for protection against enteric infection with *Citrobacter rodentium*. DOCK2 prevented or reduced bacterial attachment to enterocytes [10], as well as inhibiting macrophage migration [11]. However, these innate mechanisms seem unlikely to underlie a susceptibility to viral infections, nor do these articulate well with the known requirement for DOCK2 in lymphocytes. Here we address this gap by examining the role of DOCK2 in a well-described mouse model of herpes simplex virus (HSV) infection and antiviral T-cell immunity. HSV is not a documented problem in DOCK2 immunodeficiency, but the virus is closely related to varicella zoster virus, which is a frequent concern and lacks an animal model.

## MATERIALS AND METHODS

### Viruses and Cell Lines

HSV-1 strain KOS was kindly provided by F. Carbone (University of Melbourne) and is referred to as HSV throughout. HSV.OVA is a recombinant of HSV-1 strain KOS expressing a fusion of enhanced green fluorescent protein and the epitopes SIINFEKL, TSYKFESV, and SSIEFARL [12]. Viruses were grown and titrated by standard methods using Vero cells, which were grown in minimal essential medium supplemented with 10% fetal bovine serum (FBS), 2 mM L-glutamine, 5 × 10^−5^ M 2-mercaptoethanol (2-ME), and 5 mM HEPES (all Invitrogen).

### Mice

Specific pathogen-free C57BL/6, C57BL/6.SJL (CD45.1), and C57BL/6 OT-I mice >8 weeks of age were obtained from the Australian Phenomics Facility (APF, Canberra, Australia). DOCK2*^E775X/E775X^* (henceforth referred to as D2EX for brevity), DOCK2*^Y1307X/Y1307X^* (D2YX[1307]), and DOCK2*^Y1437X/Y1437X^* (D2YX[1437]) (ENSMUST00000093193) mice were generated by chemical mutagenesis using *N*-ethyl-*N*-nitrosourea (ENU) as previously published [13, 14]. ENU was given intraperitoneally to male C57Bl/6 mice 3 times at an interval of 1 week to generate random mutations in the DNA of spermatogonial stem cells, and offspring mice bred to bring any mutations to homozygosity. Once a phenotype and causative mutation are identified, the mice are backcrossed to C57BL/6 mice. The mutation in D2EX was identified through mapping the phenotype to an interval on proximal Chromosome 11 followed by Sanger sequencing of Dock2 as a candidate gene within the interval. The other 2 mutations were identified through exome sequencing of an affected mouse. Male and female mice were used for characterization experiments and female mice were used for all infections. All mice were housed, and experiments were done according to the relevant ethical requirements and under approvals from the ANU animal ethics and experimentation committee (A2011/01, A2013/37, A2014/62, A2016/45, A2017/54, A2020/01, A2023/09, and A2020/45) at the APF.

### HSV Infections

Female mice >8 weeks of age were anesthetized by intraperitoneal injection of Avertin (20 µL/g of body weight). The left flank of each mouse was shaved and depilated with Veet. HSV was diluted in phosphate-buffered saline (PBS) to 10^8^ plaque-forming units/mL and tattooed into a 0.5 × 0.5-cm area of skin above the tip of the spleen. Body weight and lesion progression were measured daily until the lesions had resolved. Lesion size was determined as follows. First, the overall area of skin affected by any lesions was determined with the aid of a calliper; second the proportion of the total area that had broken skin or other evidence of a herpetic lesion was estimated; finally, these 2 numbers were used to estimate a final lesion size. Lesion estimation was blinded to genotype. Dorsal root ganglia (DRG) innervating the infected dermatome were removed at day 7 postinfection, homogenized, and freeze-thawed 3 times, and viral loads were determined using a standard plaque assay [15].

### Activation and Expansion of Naive OT-I CD8^+^ T Cells by HSV Infection In Vivo

Splenocytes were prepared from D2EX and wild-type (WT) littermate CD45.1^+^ OT-I mice and enriched for CD8^+^ T cells using a MACS CD8a^+^ T Cell (untouched) Isolation Kit (#130-095-236) according to manufacturer's instructions. After purification, cells were typically approximately 90% CD8^+^, Vα2^+^; 1 × 10^4^ of these cells were injected intravenously into female CD45.2^+^ recipient mice (>8 weeks old) that were then infected on the flank with HSV.OVA 24 hours later (as above). Seven days after infection, mice were culled and numbers of OT-I cells in the spleen and/or DRG identified as CD8^+^, CD45.1^+^, Vα2^+^ events by flow cytometry.

### Activation of OT-I T Cells In Vitro

Splenocytes were prepared from D2EX and WT littermate OT-I mice. For in vitro analysis experiments, 2 × 10^6^ splenocytes were cultured with OVA_257_ peptide (SIINFEKL, concentrations as shown) in Dulbecco’s modified Eagle medium with 10% FBS, 5 × 10^−5^ M β-mercaptoethanol, and 5 mM HEPES (T-cell medium) for up to 40 hours before harvesting and flow cytometric staining for either CD69 or intracellular IRF4. For preparation of bulk cultures of OT-I T cells for transfer into mice, splenocytes were prepared as above, but cultures were started with 1 × 10^8^ splenocytes. One-third of these were pulsed with 1 × 10^−7^ M OVA_257_ peptide in serum-free medium for 1 hour at 37°C on a rocking platform before washing and recombining with the other cells. Cultures proceeded in T-cell medium, supplemented with recombinant interleukin 2 (IL-2). Cultures of D2EX OT-I failed unless supplemented with higher amounts of IL-2, and we determined empirically that using 6 ng/mL for D2EX OT-I produced adequate cultures of cells. For WT OT-I, we used 3 ng/mL of IL-2, as specified by our usual protocol. After 4 days, cultures were enriched for CD8^+^ T cells using a MACS CD8α^+^ T Cell (untouched) Isolation Kit (#130-095-236). 5 × 10^6^ purified cells (typical purity >90% CD8^+^) were transferred into female WT mice (>8 weeks old) via intravenous injection in a total volume of 200 µL PBS. Control mice received 200 µL PBS.

### Flow Cytometry

Blood was collected from the retroorbital veins using ethylenediaminetetraacetic acid as anti-coagulant. Single-cell suspensions from organs were prepared by mashing organs through a 70-µm cell strainer (BD) followed by antibody staining as described previously [16]. Erythrocytes in blood and spleen samples were lysed using ammonium chloride lysis buffer before antibody staining. Antibodies were sourced from eBioscience unless otherwise stated.

1. Peripheral blood screen: AlexaFluor700 (AF700)–conjugated anti-CD4 (BD, RM4-5), peridin-chlorophyll-protein complex (PerCP)–cyanine (Cy) 5.5 conjugated anti-B220, Pacific Blue (PB)–conjugated anti-CD44, allophycocyanin (APC)-Cy7–conjugated anti-CD3, APC-conjugated anti-NK1.1 (BD, PK136), fluorescein isothiocyanate (FITC)–conjugated anti–immunoglobulin M (IgM), phycoerythrin (PE)–Cy7 conjugated anti-KLRG1, and PE-conjugated anti–immunoglobulin D (IgD).
2. Thymic and splenic stains: AF700 conjugated anti-CD4 (BD, RM4-5), Brilliant Ultraviolet (BUV) 395-conjugated anti-CD8 (BD, 53-6.7), APC-conjugated anti-CD5 (53–7.3), PE-conjugated anti-CD25 (PC61.5), PerCP Cy 5.5-conjugated anti-CD3 (BioLegend, 17A2), PE-conjugated anti-CD3 (BD, 145-2C11), Brilliant Violet (BV) 605 conjugated anti-CD62L (BioLegend, MEL-14), PB-conjugated anti-CD44 (BioLegend, IM7), APC-Cy7-conjugated live/dead stain, FITC-conjugated anti-TCR-β (H57-597, eBioscience), efluoro-conjugated live/dead stain, biotin-conjugated anti-CD93 (AA4.1), PE-Cy7-conjugated IgM (II/41), FITC-conjugated anti-IgD (11-2c (22–26), PB-conjugated anti-CD23 (B3B4), BUV737-conjugated anti-CD21/35 (BD, 7G6), AF700-conjugated anti-B220 (RA3-6B2) and BUV395-conjugated anti-CD19 (BD, 1D3). All B-cell stains included Fc block (BD, 2.4G2), either as a 30-minute preincubation or together with biotinylated or fluorescently labeled antibodies. Biotin staining was followed by addition of BV605-conjugated streptavidin (BioLegend). For intracellular staining of FOXP3, the eBioscience Foxp3/Transcription Factor Staining buffer set (00-5523-00) was used with FITC-conjugated anti-FoxP3 antibodies (FJK-16S). Detection of natural killer T (NKT) cells using CD1d monomers loaded with α-GalCer (produced by the National Institutes of Health tetramer facility) was performed as described previously [17].
3. For CD8^+^ cells and HSV-specific CD8^+^ T cells in infected mice, the panel included H-2Kb/SSIEFARL dextramer (Immudex), anti-CD8 (clone 53-6.7; BioLegend), and in some cases anti-CD62L (MEL-14, BioLegend) and intracellular staining with anti-granzyme B (GzmB) (GB11, BioLegend). After stimulation with gB_498–505_ peptide (hereafter referred to as gB_498_ or by its sequence, SSIEFARL) for 4 hours in the presence of brefeldin A, anti-CD8 (as above) and anti–interferon gamma (IFN-γ) (XMG1.2, BioLegend), stained intracellularly [18]. For OT-I cells tested prior to transfer or from mice after transfer and infection, anti-CD8 (as above), anti-CD45.1 (A20, BioLegend) and anti-TCRVα2 (B20.1, BioLegend). For OT-I cells stimulated in vitro, anti-CD8 (as above) and anti-CD69 (HI.2F3, BD Bioscience) or anti-IRF4 (3e4, eBioscience) stained using a Transcription Factor Staining Buffer Set (catalog number 00-5523-00, eBioscience).

### Migration Assay

CD8^+^ T cells were negatively sorted (CD19^−^, CD4^−^, CD11b^−^, NK1.1^−^) on a BD FACS Aria II or FACS FUSION from OT-I spleens, resuspended to a concentration of approximately 1 × 10^6^/100 μL of low serum (0.5% bovine serum albumin) RPMI media, and plated on the top well of a Corning Transwell plate (Merck, 5.0 μm pore size). Six hundred microliters of media was added to the lower chamber containing the chemoattractant (150 ng/mL CXCL12, Pepro Tech), or media alone. Cells were incubated at 37°C and 5% carbon dioxide and allowed to migrate for 3.5 hours. Migrated cells were counted using absolute counting beads (CountBright, Thermo Fisher) for flow cytometry.

## RESULTS

### Novel DOCK2 Mutant Mouse Strains

**Figure 1.**
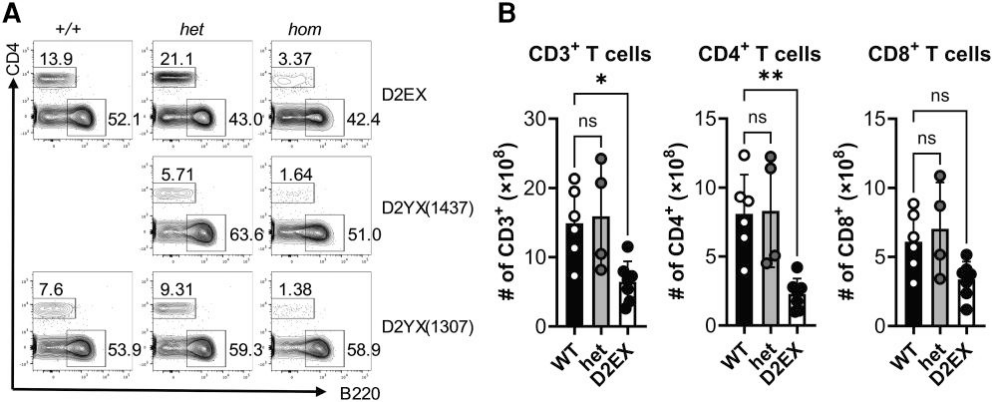
T-cell lymphopenia in the absence of DOCK2. *A*, Representative flow cytometry plots (pre-gated on lymphocytes) from mice carrying 2 copies (*hom*), 1 copy (*het*), or no copies (*+/+*) of the listed amino acid change in DOCK2. *B*, Quantification of CD3^+^, CD4^+^, and CD8^+^ T cells in the blood of wild-type (WT), D2EX heterozygous, and D2EX homozygous mice. Data from 1 experiment with 4–6 mice per genotype. One-way analysis of variance with Dunnett multiple comparisons test. *P < .05, **P < .005; ns, not significant.

As part of an ENU-mutagenesis project to provide mouse models for human disease [13], 3 different mouse strains with premature stop codons in *DOCK2* were discovered due to T-cell lymphopenia in the blood as shown in Figure 1*A*. The position of the mutations in the DOCK2 protein are shown in Supplementary Figure 1*A*. The first strain identified had a premature stop codon (E775X mutation due to a G to T point mutation at position 2392 in cDNA [ENSMUST00000093193]) and was analyzed further. Homozygous mice carrying this mutation (ie, DOCK2*^E775X/E775X^*) are referred to hereafter as D2EX for brevity and recapitulate published features of DOCK2-deficient mice, whereas heterozygotes have a WT phenotype. These features include marked T-cell lymphopenia [7] in the blood despite a normal total number of leukocytes (Figure 1*A* and 1*B*, Supplementary Figure 1*B* and 1*C*). Marginal zone B cells in secondary lymphoid organs are absent [7] and NKT cells are decreased in the thymus [19] (Supplementary Figure 1*D* and 1*E*). We also detected elevated levels of eosinophils and immunoglobulin E with aging in these mice, suggesting they may be prone to allergy (Supplementary Figure 1*C* and data not shown).

**Figure 2.**
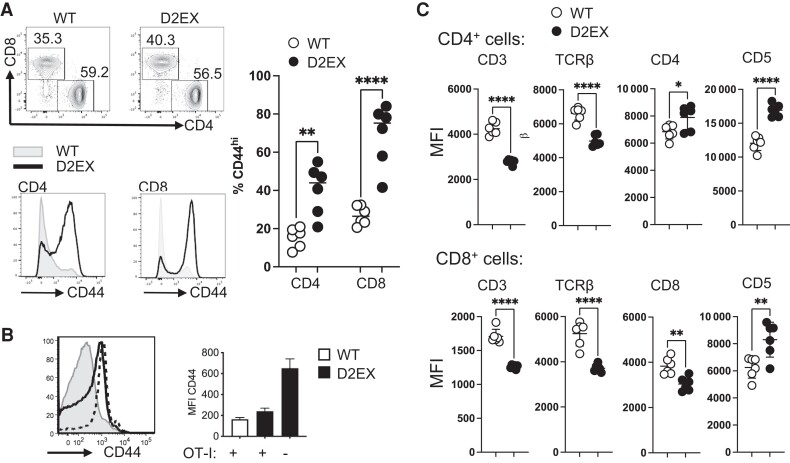
Altered activation of T cells in the spleen of D2EX mice. *A*, Representative flow cytometry plots (pre-gated on CD3^+^ lymphocytes) from wild-type (WT) and mutant mice (top panel) and quantitation of proportion of CD44^hi^ CD4^+^ and CD8^+^ T cells from the 2 groups (right panel). Representative histograms showing the CD44 staining of CD4^+^ and CD8^+^ T cells from WT (gray) and mutant mice (black line). Representative of at least 3 separate experiments. *B*, Effect of limiting T-cell receptor repertoire on the expansion of CD44^+^ lymphocytes. Representative histogram of CD44 expression for WT mice with OT-I (gray), D2EX mice with OT-I expression (dotted), and D2EX mice without OT-I (black line), and quantitation of mean fluorescence intensity (MFI) for these groups of mice—wild-type mice with OT-I (white bar), D2EX mice with OT-I expression (black bar), and D2EX mice without OT-I (black bar) with absence/presence of OT-I noted on the x-axis. *C*, Relative expression of the listed markers on wild-type and mutant CD4^+^ (upper panel) and CD8^+^ (lower panel) T cells. Representative of at least 2 separate experiments. Unpaired *t* test. *P < .05, **P < .005, ****P < .0001.

Closer analysis of T-cell subsets in the spleen of these mice shows that the majority of the T cells (both CD4 and CD8) have an activated phenotype marked by high expression of CD44 (Figure 2*A*), as described previously [9]. However, when the D2EX mutation was bred onto OT-I mice, in which all CD8^+^ T cells have the same TCR and so cannot respond to a normal range of self-antigens, this activation phenotype was partially ameliorated (Figure 2*B*).

Average expression of CD3 and TCRβ, both of which are components of the TCR and are required for responses to antigen, were decreased on D2EX CD4^+^ and CD8^+^ T cells. Also in line with dysregulated responses to antigen we find that CD5 expression, which is positively correlated with TCR signal strength [20, 21], is increased on T cells in the spleen (Figure 2*C* and Supplementary Figure 2). Expression of the CD8 co-receptor on CD8^+^ T cells was decreased but conversely, expression of CD4 was increased on mutant CD4^+^ T cells. These features also align with published DOCK2-deficient phenotypes [9].

**Figure 3.**
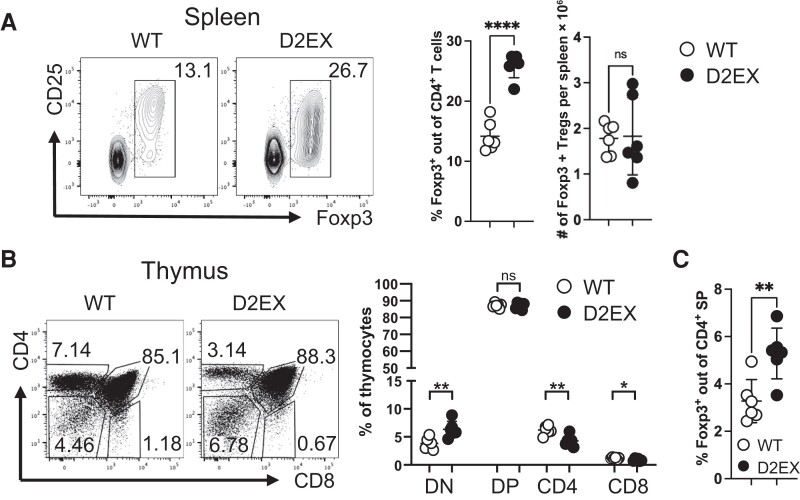
Increased formation of Foxp3^+^ Tregs in the absence of DOCK2. *A*, Naive mice were analyzed by flow cytometry for the % and number of splenic Foxp3^+^ Tregs. *B*, Thymic Tcell development was analyzed in naive mice. *C*, Thymic Foxp3^+^ cells were increased as a percentage of CD4 single positive T-cells. Unpaired *t* test. *P < .05, **P < .005, ****P < .0001. Data representative of 3 independent experiments. Abbreviations: DN, double negative; DP, double positive; SP, single positive; WT, wild type.

We also enumerated regulatory T cells (Tregs) in the spleen based on expression of FoxP3 and found that the percentage was increased in D2EX mice (Figure 3*A*). Considering the peripheral T-cell lymphopenia noted above, thymic T-cell subsets in D2EX mice showed more modest differences compared to WT littermates (Figure 3*B*). However, consistent with the increase of Tregs in the spleen, there was a proportional increase in FoxP3^+^ cells within the CD4^+^ subset in the thymus (Figure 3*C*). Finally, thymic NKT cells were reduced (Supplementary Figure 1*E*), further confirming that D2EX mice recapitulate a broad range of cellular defects previously described for DOCK2 mutants [19].

### D2EX-Mutant Mice Have Impaired Control of HSV-1

**Figure 4.**
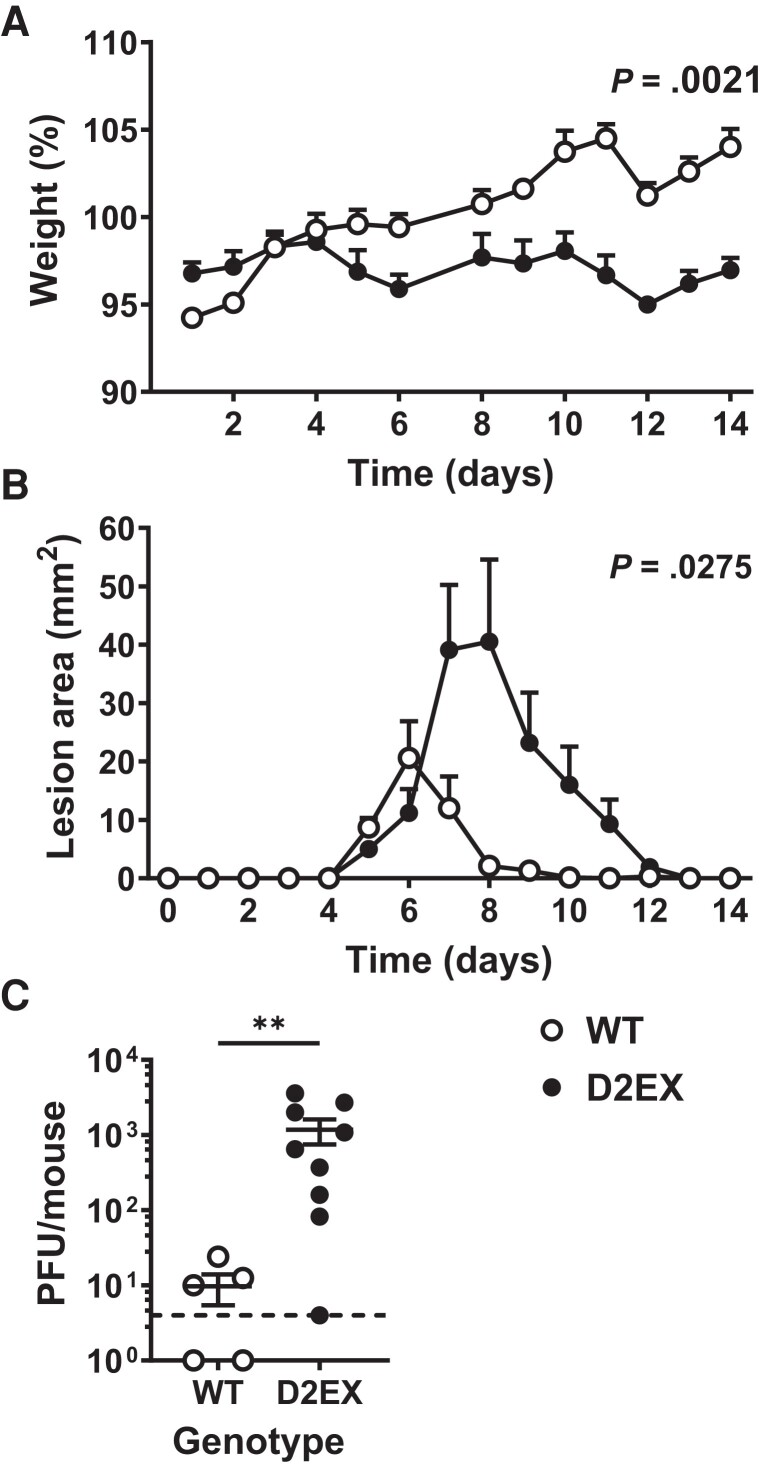
Delayed clearance of herpes simplex virus (HSV) in the absence of DOCK2. Mice were infected with HSV on the flank, and pathogenesis and viral loads were measured. Weights (*A*) and lesion sizes (*B*) of groups of 6 wild-type (WT) and 7 D2EX mice were monitored for 14 d. Differences between the strains were determined by 2-way analysis of variance, with significant *P* values noted in the top right of graphs. *C*, Loads of infectious virus (plaque-forming units [PFU]) in the dorsal root ganglia of mice were measured by plaque assay 7 d after infection and the difference in means was tested using *t* test (**P < .01). The experiment in *A* and *B* is representative of 3 independent repeats. *C* shows data combined from 2 independent experiments.

To test their ability to respond to virus infection, groups of D2EX and WT mice were inoculated with HSV in the flank. In this model, infection begins in the inoculated skin before spreading to the peripheral nervous system and then back to additional sites in the skin [22, 23]. After HSV infection, all mice lost weight briefly, but D2EX mutants were slower to regain weight compared with WT littermates (Figure 4*A*). Furthermore, D2EX mice developed significantly larger lesions that took longer to resolve than those on WT mice (Figure 4*B*). Using the same model, we found that D2EX mice had significantly higher viral loads than WT mice in DRG 7 days after infection (Figure 4*C*). We conclude that D2EX mice are ultimately able to clear disease and therefore infectious HSV, but clearance is impaired, or delayed [22, 23].

### DOCK2 Has a Cell-Intrinsic Role in Priming Antiviral CD8^+^ T-Cell Responses

**Figure 5.**
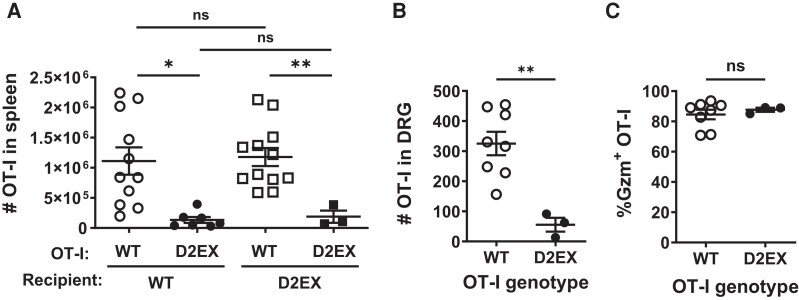
DOCK2 plays a cell-intrinsic role in clonal expansion of antiviral CD8^+^ T cells. CD8^+^ T cells purified from OT-I mice of the genotypes shown were transferred into wild-type (WT) and D2EX (*A*) or WT (*B* and *C*) mice that were then infected with HSV.OVA 24 h later. *A*, Numbers of OT-I T cells in the spleens of mice 7 d after infection; data were combined from an initial experiment with all 4 groups and 2 further experiments including all groups except D2EX mice transferred with D2EX OT-I. Numbers of OT-I T cells in the dorsal root ganglia (DRG), 7 d after infection from 2 experiments (*B*) and the percentage of these cells expressing granzyme B (GzmB) (*C*). Statistical significance was determined using a 2-way analysis of variance followed by Sidak posttest for pairwise comparisons (*A*) or *t* tests (*B* and *C*); *P < .05, **P < .01; ns, not significant.

HSV infection of mice has provided an excellent model for interrogating CD8^+^ T-cell immunity, which is relevant to human infections with this virus [24–30]. To examine the role of DOCK2 in CD8^+^ T cells, we used D2EX mice bred onto the OT-I TCR-transgenic, in which all CD8^+^ T cells have a TCR that recognizes the SIINFEKL peptide. Equal numbers of CD8^+^ T cells from WT and D2EX OT-I mice were transferred into regular WT and D2EX mice, which were then infected with HSV.OVA, which expresses SIINFEKL, and their expansion was measured after 7 days. Irrespective of the recipient genotype, WT OT-I cells expanded in response to infection such that an average of approximately 1 × 10^6^ were found in the spleen. By contrast, D2EX OT-I cells failed to expand well, with around 10-fold fewer being found (Figure 5*A*). This significant difference in OT-I number was also seen in DRG, but surprisingly GzmB expression, which is a marker of cytotoxic function, was similar for both genotypes (Figure 5*B* and 5*C*). These data suggest that DOCK2 has a cell-intrinsic role in CD8^+^ T-cell priming and expansion, and possibly in migration to infected sites.

### DOCK2 Is Required for the Full Protective Effect of Antiviral CD8^+^ T Cells

**Figure 6.**
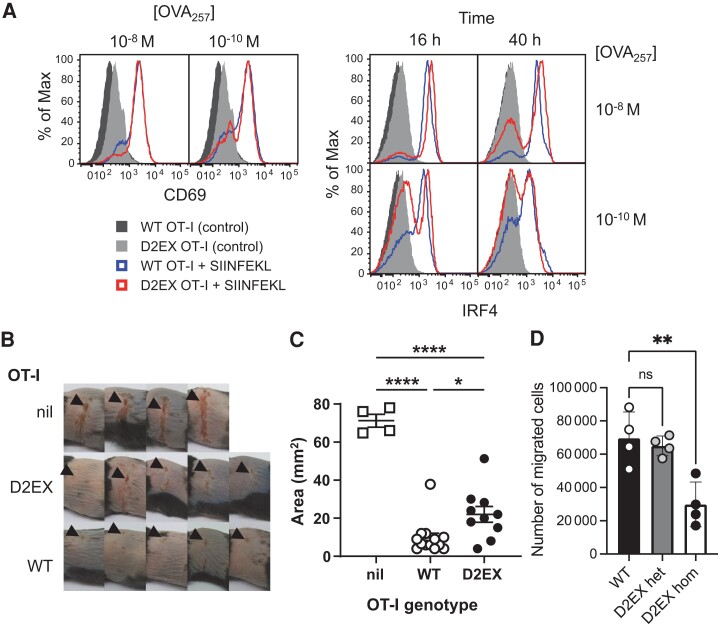
DOCK2-deficient CD8^+^ T cells have slightly reduced protective capacity against herpes simplex virus (HSV) infection. CD8^+^ T cells purified from OT-I mice of the genotypes shown were activated with peptide in vitro. *A*, CD69 and IRF4 were measured at 24 h and at 16 and 40 h, respectively. *B* and *C*, OT-I CD8^+^ T cells were primed and expanded for 4 d and then transferred into wild-type (WT) mice that were infected with HSV.OVA 24 h later. Images of lesions on mice (*B*) and peak lesion areas (*C*) are shown compared with mice that received no cells (nil). Data were combined from 2 independent experiments; points represent individual mice with bars showing mean and standard error of the mean. *D*, Transwell migration assay of OT-I CD8 T cells in response to CXCL12. Statistical significance was determined by 1-way analysis of variance with Sidak posttest for pairwise comparisons; *P < .05, **P < .01, ****P < .0001; ns, not significant.

To examine the priming defect in D2EX OT-I cells, these were cultured for 24 hours in the presence of SIINFEKL peptide to prime them in vitro. We were surprised to find that initial TCR signaling, as indicated by upregulation of CD69 as an early activation marker, was the same for WT and D2EX OT-I cells, even under limiting peptide stimulation (Figure 6*A*, left). However, IRF4 expression, which indicates the adequacy of priming and predicts clonal expansion [12] clearly differed, with only WT OT-I strongly upregulating IRF4 by 16 hours and maintaining much of this expression across the population for 40 hours (Figure 6*A*, right). This was especially notable when antigen was limiting at 10^−10^ M.

Next, to determine whether D2EX CD8^+^ T cells might have antiviral function if adequate numbers can be primed, we activated D2EX and WT OT-I cells in vitro, then transferred equal numbers of these into groups of WT mice before infection with HSV.OVA. Surprisingly, activated D2EX OT-I cells provided significant protection from lesions caused by HSV.OVA. (Figure 6*B* and 6*C*, Supplementary Figure 3). However, there was a statistically significant difference in the protection provided by WT and D2EX OT-I cells as determined by peak lesion area, with WT cells being superior.

Previous studies [7] have shown a role of DOCK2 for cell migration, which could contribute to the reduced effectiveness of the transferred D2EX OT-I T cells. To test this, we performed a transwell migration assay demonstrating a reduced migration capacity of DOCK2-deficient CD8 T cells (Figure 6*D*). Taken together, we conclude that the major defect in D2EX CD8^+^ T cells is in adequate expansion, with a more modest reduction in the ability of primed cells to counter infection, most likely linked to impaired migration.

### Reduced Endogenous Virus-Specific CD8^+^ T Cells in D2EX Mice

**Figure 7.**
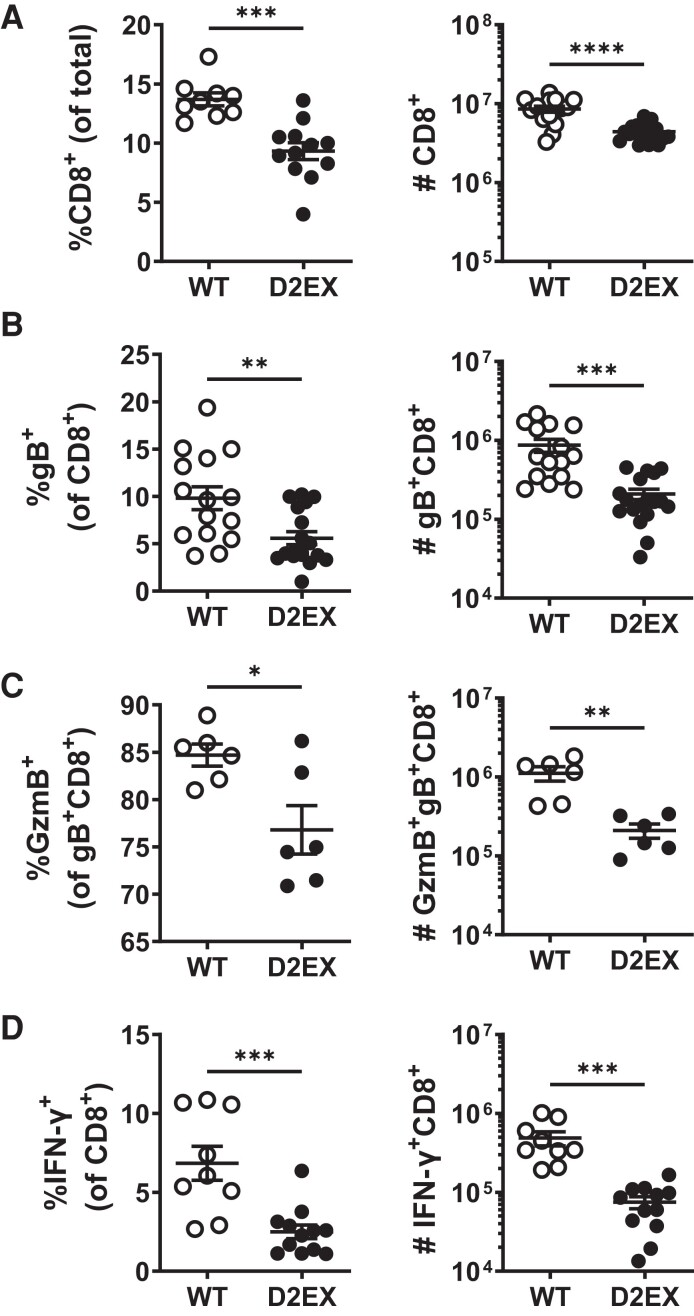
CD8^+^ T-cell responses to herpes simplex virus (HSV) are deficient in the absence of DOCK2. Mice were infected with HSV and various attributes of CD8^+^ T cells were measured in spleens 7 d later. Graphs on the left and right of each panel show the percentages and total numbers of the populations shown, respectively. All CD8^+^ T cells (*A*), HSV-gB_498_-specific CD8^+^ T cells (*B*), granzyme B (GzmB)^+^, gB_498_-specific CD8^+^ T cells (*C*), and CD8^+^ T cells (*D*) were able to make interferon gamma (IFN-γ) after stimulation with gB_498_ peptide. Data shown are combined from 5 (*A*, *B*), 3 (*D*), and 2 (*C*) independent experiments. Statistical significance was determined by *t* tests; *P < .05, **P < .01, ***P < .001, ****P < .0001.

To test whether the priming of nontransgenic CD8^+^ T-cell responses to HSV might be affected in D2EX mice, we examined these cells in the spleens 7 days after infection. Just as in uninfected mice, the percentage and total number of CD8^+^ T cells were lower in D2EX mice than in WT controls (Figure 7*A*, Supplementary Figure 4). CD8^+^ T cells with a TCR specific for the dominant epitope of HSV (gB_498_; SSIEFARL) were also reduced in D2EX mice, and fewer of these cells were expressing GzmB (Figure 7*B* and 7*C*). Finally, the percentage and total number of CD8^+^ T cells able to make IFN-γ in response to stimulation with SSIEFARL peptide was also reduced in D2EX, compared with WT mice (Figure 7*D*). We also examined CD8^+^ T cells in the skin of infected mice and found that there were fewer total CD8^+^ T cells and gB_498_-specific, CD8^+^ T cells in D2EX compared with WT mice (Supplementary Figure 5). Taken together, findings from an analysis of the endogenous CD8^+^ T-cell response are largely consistent with those gained with transferred OT-I T cells.

## DISCUSSION

DOCK2 deficiency has been well studied in uninfected mice, and our new DOCK2 mouse strains replicate these findings with absent marginal zone B cells [7], low numbers of NKT cells [19], and T-cell lymphopenia [7]. Likewise, we also observed the apparent “activation” of DOCK2-defective T cells with increased CD44 expression [9], but we add that this is partially overcome in mice with a limited T-cell repertoire (OT-I mice). We also show that DOCK2 mice have eosinophilia on a C57BL/6 background whereas previously this was only shown in TH2-prone Balb/c mice [31]. Another new finding is the sparing of Treg (FoxP3^+^) cells with these being present at a higher proportion than other CD4^+^ T-cell subsets, which might be due to altered TCR signal strength (reflected here also by CD5 levels) that may favor Treg production [32]. This contrasts with a previous report that found DOCK2 deficiency might be linked to the production of fewer FoxP3^+^ or IL10^+^ Tregs, but this was done using in vitro cultures [33].

DOCK2-deficient patients have an increased susceptibility to viruses and this has been ascribed to defects in either T cells or NK cells without a further dissection of mechanisms [34]. We show that DOCK2 is important for the control of HSV and also for the normal function of anti-HSV T cells. Interestingly, initial in vitro activation of the mutant T cells by expression of CD69 was normal despite the previously found defect in synapse assembly [5]. However, poor expression of IRF4 shows that this engagement is suboptimal in the absence of DOCK2 and, ultimately, expansion of the virus-specific CD8^+^ T cells fails. The finding that once primed, DOCK2-deficient T cells can retain significant protective function was somewhat surprising given previous in vitro data on synapse defects. At the same time, this antiviral activity was reduced compared to DOCK2-sufficient cells, likely due in part to the poor migration of these cells.

Two limitations of our study require noting. First, while we highlight defects in CD8^+^ T-cell immunity, we have not examined other aspects of the antiviral response. In particular, the defects in antiviral CD8^+^ T-cell priming seen here might similarly affect antiviral CD4^+^ T cells. In this regard, we note that DOCK8 has many overlapping function with DOCK2, and mice lacking DOCK8 have been found to have defects in both T-cell subsets in response to HSV [15, 35]. Also as noted above, there are relatively more Treg cells in D2EX mice, which might impede immunity. Second, we have only examined HSV here, so it remains to be seen whether similar defects in antiviral defence will be found for other viruses.

In summary, we show here that DOCK2 deficiency leads to reduced control of HSV infections in mice and that this is associated with impaired T-cell immunity.

## Supplementary Data

Supplementary materials are available at *The Journal of Infectious Diseases* online (http://jid.oxfordjournals.org/). Supplementary materials consist of data provided by the author that are published to benefit the reader. The posted materials are not copyedited. The contents of all supplementary data are the sole responsibility of the authors. Questions or messages regarding errors should be addressed to the author.

## Supplementary Material

jiae077_Supplementary_Data

## Notes

***Acknowledgments.*** We thank the National Computational Infrastructure (Australia) for continued access to significant computation resources and technical expertise; the staff of the Australian Phenomics Facility for animal husbandry; the staff of Australian Cancer Research Foundation Biomolecular Resource Facility for DNA preparation, genotyping, exome and Sanger sequencing; and the staff of the Microscopy and Cytometry Resource Facility for help with flow cytometry.

***Financial support*.** This study was funded by the National Institutes of Health (NIH) (grant number U19-AI100627); the Australian National Health and Medical Research Council through project grant 1144684 to A. E. and K. L. R. and fellowship 1104329 and investigator grants 2008990 to D. C. T.; Australian Research Council DP190101325 to D. C. T.; and Australian Capital Territory Private Practice Major Grant 2015 and 2016 to K. L. R. CD1d tetramers used in this study were provided by the NIH tetramer facility (Bethesda, Maryland).
