# Supplementary material for: DOCK2 Deficiency Causes Defects in Antiviral T-Cell Responses and Impaired Control of Herpes Simplex Virus Infection
Source: J Infect Dis. 2024 Feb 15;230(3):e712–21. doi: 10.1093/infdis/jiae077 (PMC11420714; doi:10.1093/infdis/jiae077)
Supplement: jiae077_Supplementary_Data [file jiae077_supplementary_data.pdf]

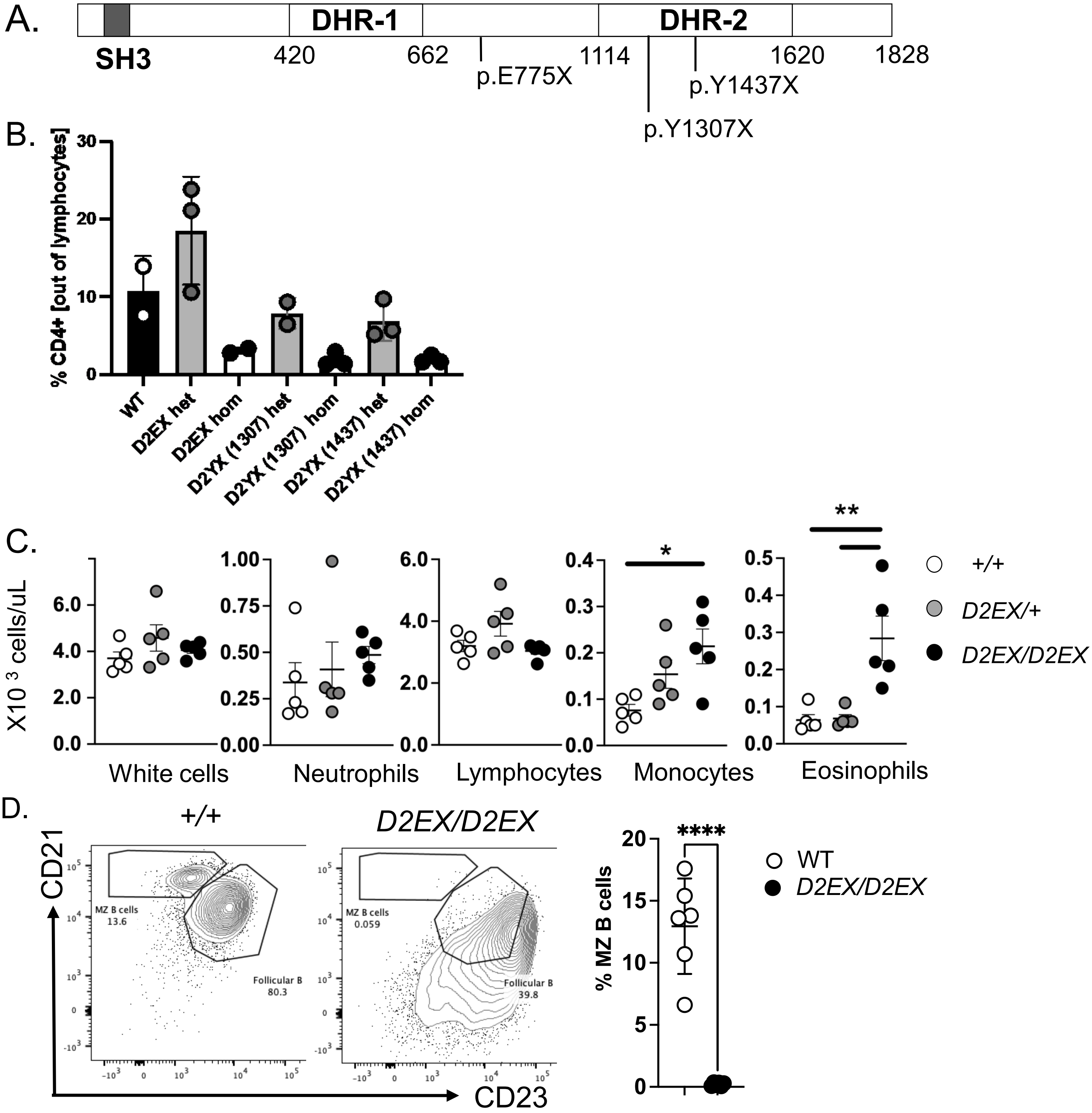

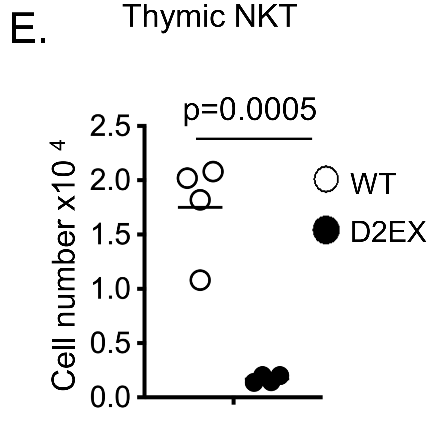

Supplementary Figure 1:

A. Graphical representation of the DOCK2 protein structure indicating the sites of the ENU-induced mutations
B. Quantification of CD4+ T cells in the blood of mice of the indicated genotype. Data is from the same experiment as shown in Figure 1A.
C. Quantification of the white cell count and differential in the peripheral blood for wild type, heterozygous and mutant mice. Statistical analysis by one way ANOVA with Tukey's multiple comparison test. *<0.05, **<0.005
D. Representative flow cytometry plots (left) and quantitation (right) of marginal zone B cells in the spleen of wild type (white) and mutant (black) mice. Statistical analysis was done by unpaired t-test. ****p<0.0001.
E. Number of thymic NKT cells. Dots represent individual mice, and the mean is indicated by the bar. Statistical analysis by unpaired t –test.

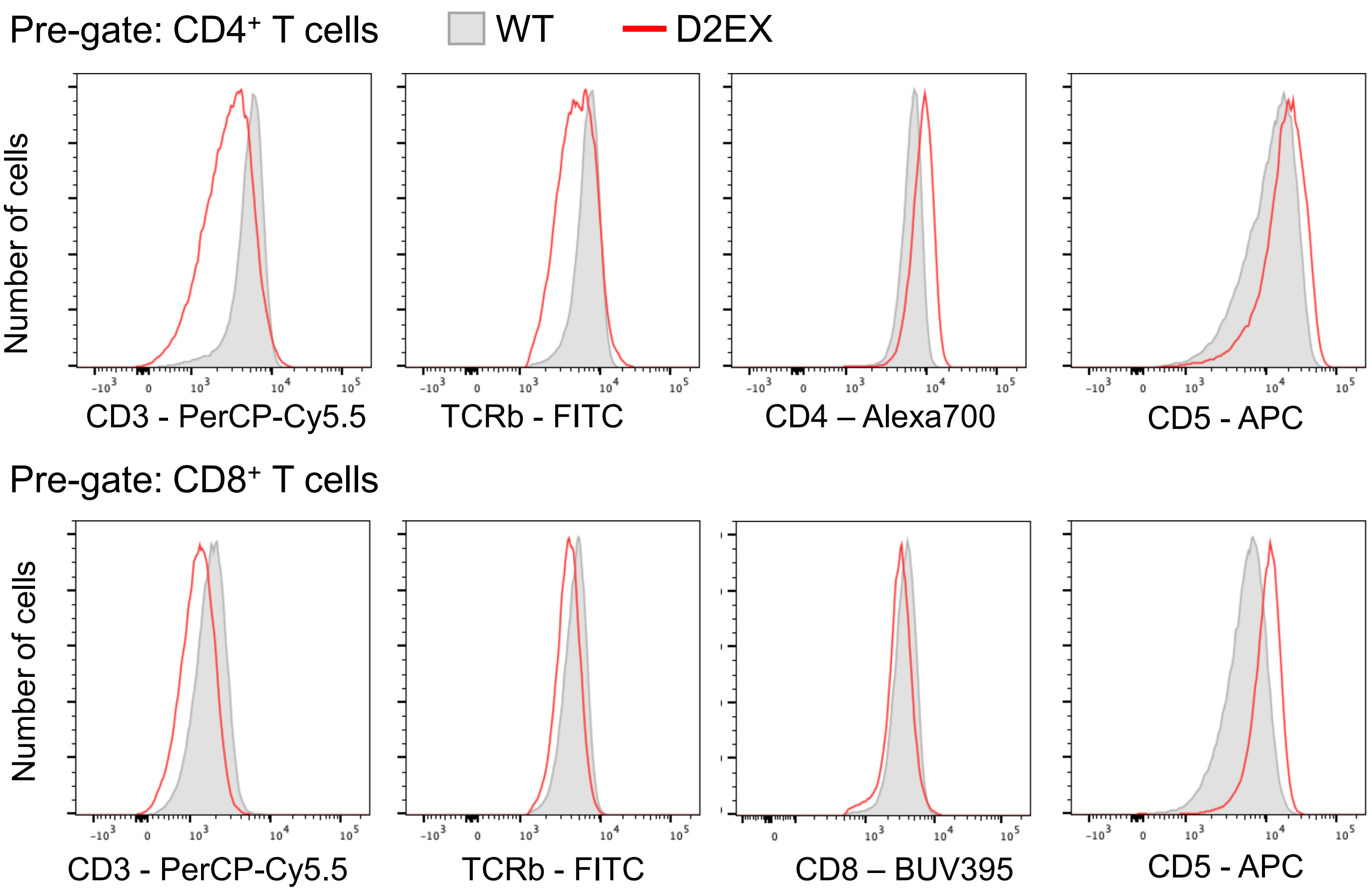

Supplementary Figure 2. Altered surface marker expression on D2EX T cells. Representative histogram overlays for CD4+ (top) and CD8+ (bottom) T cells from the spleen of wild-type and D2EX mice.

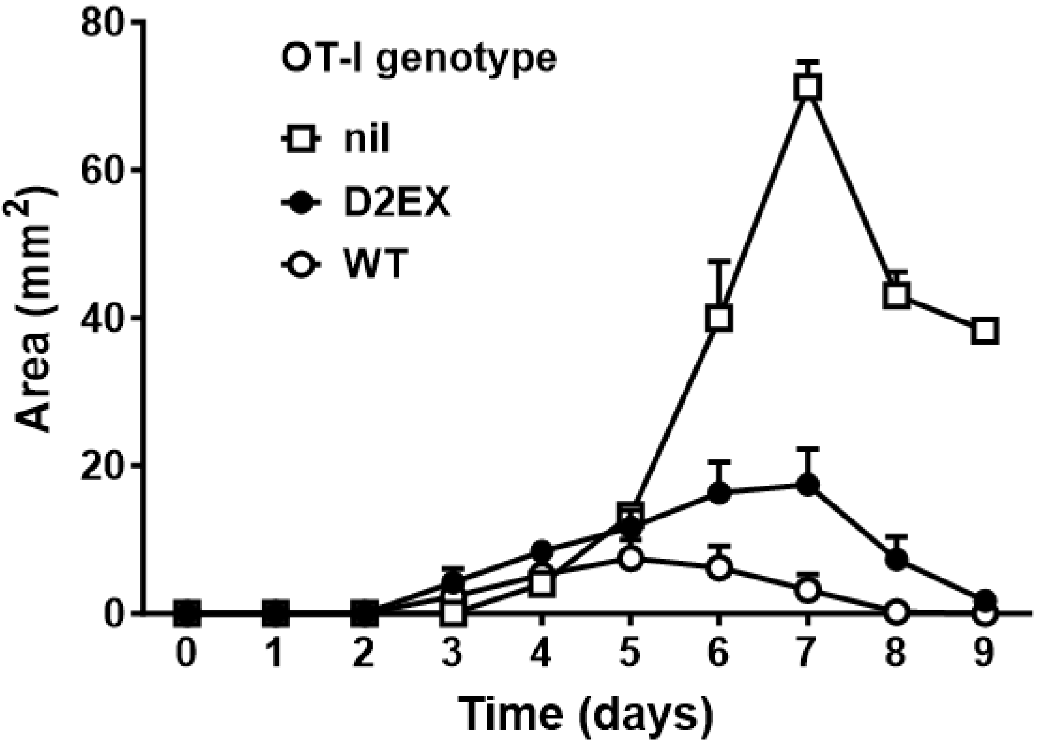

Supplementary Figure 3. Progression of lesions following HSV infection after transfer of OT-I T cells. Estimated lesion area over time in B6 mice that received $5x10^6$ activated OT-I T cells of the indicated genotype (or no cells, nil) 24 hr before tattoo-infection with HSV.OVA pC-GIP. Combined data are shown from two independent experiments, each with 4-7 mice per group.

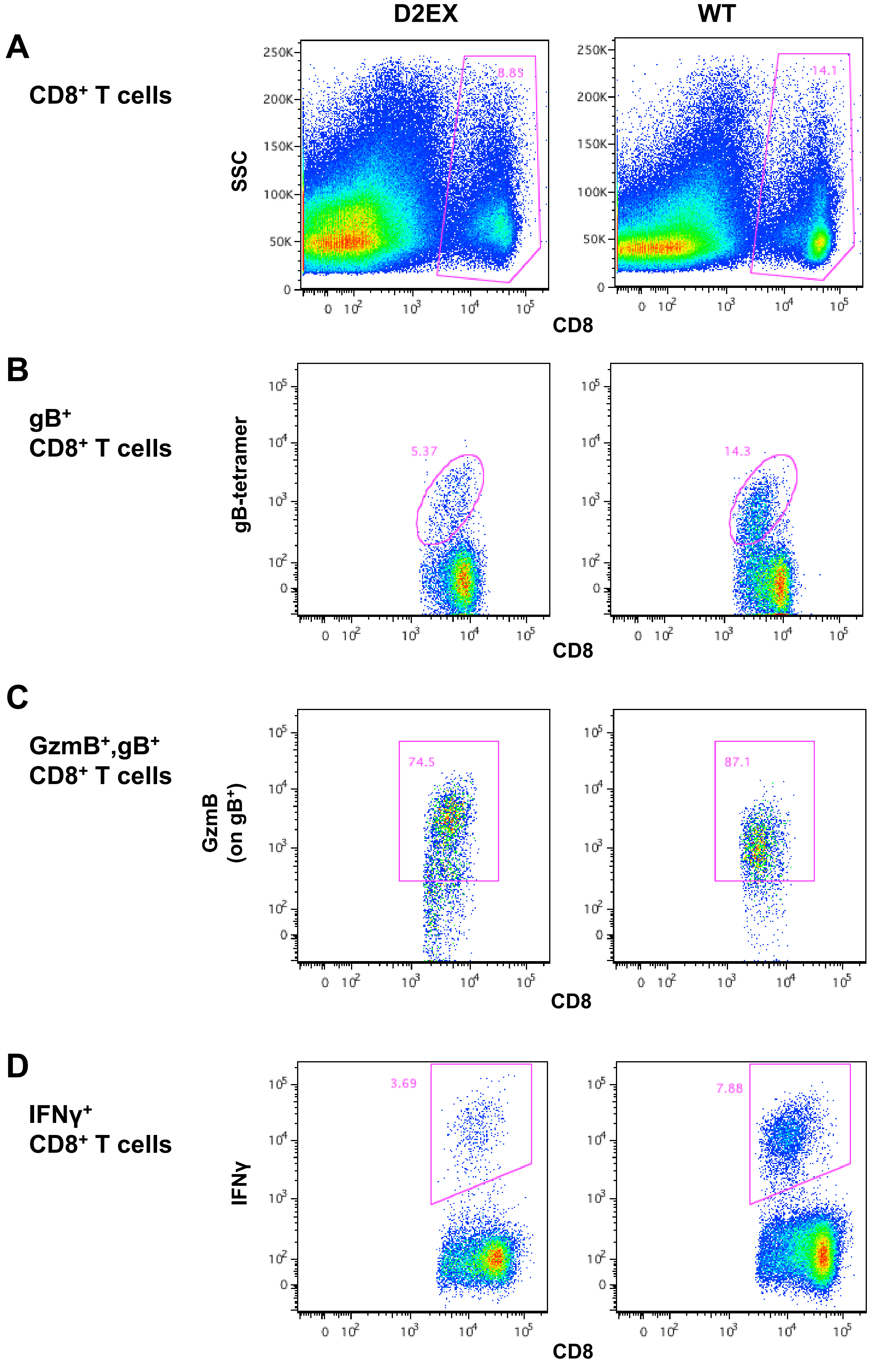

Supplementary Figure 4: Representative flow cytometry plots for the summary data shown in Figure 7; Plots from D2EX and WT mice are on the left and right, respectively.

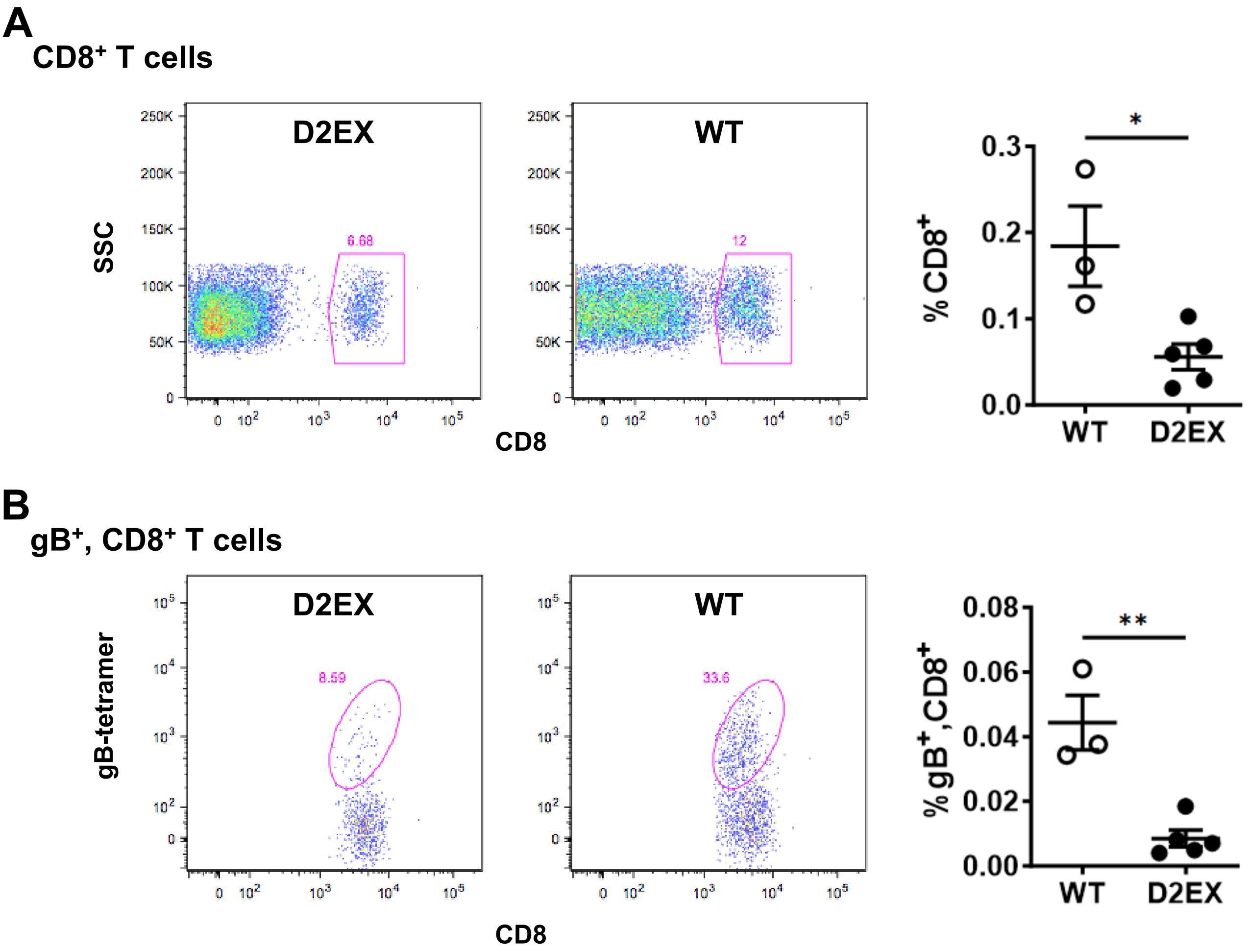

Supplementary Figure 5: CD8⁺ T cells and gB⁺, CD8⁺ T cells in the skin of WT and D2EX mice infected with HSV. Representative flow cytometry plots on the left and summary figures showing fraction of total skin infiltrating cells for each population on the right.
